# Evolutionary Divergence between *Toona ciliata* and *Toona sinensis* Assayed with Their Whole Genome Sequences

**DOI:** 10.3390/genes13101799

**Published:** 2022-10-05

**Authors:** Xi Wang, Yu Xiao, Zi-Han He, Ling-Ling Li, Yan-Wen Lv, Xin-Sheng Hu

**Affiliations:** 1 College of Forestry and Landscape Architecture, South China Agricultural University, Guangzhou 510642, China; 2 Guangdong Key Laboratory for Innovative Development and Utilization of Forest Plant Germplasm, Guangzhou 510642, China

**Keywords:** Meliaceae, *Toona ciliata*, *Toona sinensis*, whole genome duplication, long-terminal repeats, genome sequence

## Abstract

*Toona ciliata* and *Toona sinensis* belong to the *Toona* genus of the Meliaceae family and are important timber species in China. *T. ciliata* is an endangered species at level II due to overcutting and a low rate of natural regeneration. *T. sinensis* was cultivated as an economic and nutritious tree for more than 2000 years. The two species differ in flower and leaf morphological traits, reproductive systems, and range size of natural distribution. To reveal the potential molecular basis of these divergences, we examined the similarities and differences in their whole genome sequences. Results indicate that *T. ciliata* had a higher number of expanded gene families than *T. sinensis*. The whole genome duplication (WGD) occurred before their speciation. The long-terminal repeats (LTRs) insertion was earlier in the *T. ciliata* genome (3.2985 ± 2.5007 Mya) than in the *T. sinensis* genome (3.1516 ± 2.2097 Mya). Twenty-five gene families in the *T. ciliata* genome were detected to be under positive selection compared with background branches of ten different land species. The *T. ciliata* genome was highly collinear with the *T. sinensis* genome, but had low collinearity with the genomes of more distant species. These genomic and evolutionary divergences are potentially associated with the differences between *T. ciliata* and *T. sinensis* in terms of their reproductive systems and ecological adaptation.

## 1. Introduction

*T. ciliata* and *T. sinensis*—both also known as Chinese mahogany—are deciduous or semi-deciduous tree species in the *Toona* genus of the Meliaceae family [1]. Both species are important timber species in Southern China. *T. ciliata* is mainly distributed in southeast Asia, in countries such as India, Malaysia, and Indonesia. It is distributed in southern China at 200–1000 m above sea level. Its wood is tough and of straight texture, with beautiful patterning, dark reddish-brown heartwood, and light sapwood. It is an ideal material for art processes and antique furniture, and is a precious wood species in China, with a significant economic value [2]. *T. ciliata* is an endangered species at level II due to overcutting and a low rate of natural regeneration [3]. Inbreeding depression occurs in natural forests of *T. ciliata* [4].

*T. sinensis* is mainly distributed in subtropical and tropical regions in China and it grows below 1500 m above sea level [1]. It was cultivated as a precious economic tree in China for more than 2000 years [5]. It has multi-functional purposes as a high-quality wood material and as a medicinal plant [6,7]. The species contains flavonoids that are commonly used to treat bacterial dysentery, pulmonary cough, hematochezia, enteritis, and other diseases [8]. In general, *T. sinensis* has considerable value as a nutritious, medicinal plant and as a timber species [1,9].

**Figure 1:**
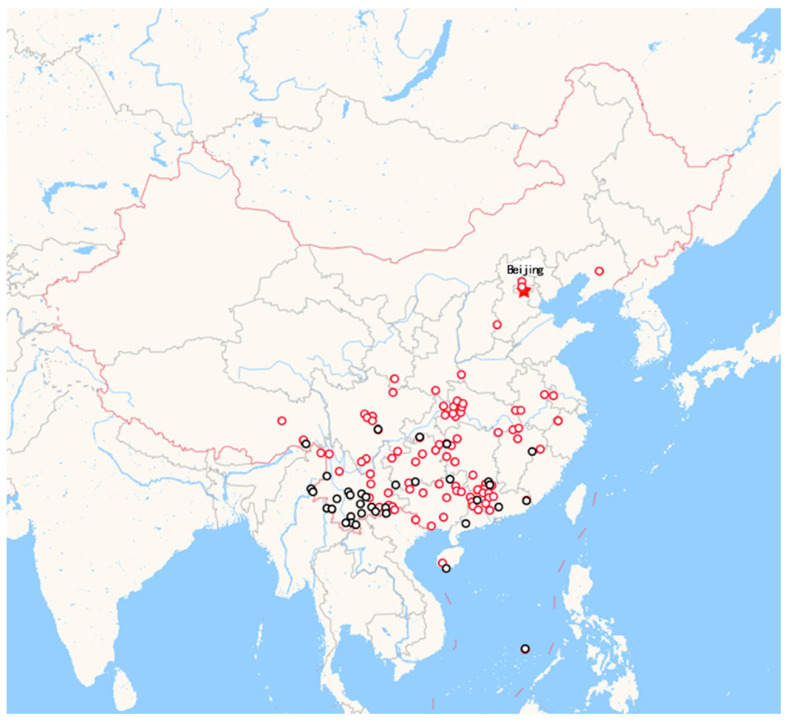
Different ranges of natural distributions of *T. ciliata* (black circles) and *T. sinensis* (red circles) in China. This figure was drawn by synthesizing the distribution maps of specimens of *T. sinensis* and *T. ciliata* from the Chinese Virtual Herbarium website. Distribution of each species can be searched by inputting the species names on the website. Each point represents a record of specimens collected from the corresponding position. The red star represents the position of Beijing, the capital city of China.

The two species are identified in a taxonomy based on their leaf and flower morphological traits [1]. They differ substantially in natural distribution and ecological adaptation. *T. ciliata* has a much narrower range of natural distribution than *T. sinensis* and is embedded within the natural distribution of *T. sinensis* in China (Figure 1). One well-known difference is that the young leaves of *T. sinensis* are edible and nutritious [10,11]; however, the young leaves of *T. ciliata* are not edible, implying that distinct metabolic chemicals or pathways could be involved in the biosynthesis of some unknown nutritious components. In addition, the two species have distinct reproductive systems. *T. sinensis* is functionally sexually diecious and possesses a complete outcrossing system; *T. ciliate,* in contrast, retains perfect flowers and possesses a predominant outcrossing system, with partial selfing and inbreeding [12]. These differences could be related to their underlying differences in genetic basis and are associated with their genomic divergences formed through long-term evolutionary processes.

Although the two species are sympatric, natural or artificial hybridization is rarely reported in the literature. Their different ranges of natural distribution in China imply that *T. sinensis* is adaptive to more diverse habitats than *T. ciliata*. Natural selection could be involved in forming their divergence in geographical distribution. Based on the sequences of nuclear ITS and cpDNA segments (*trn*S-*trn*G, *psb*B, *psb*T, and *psb*N genes), Muellner et al. [13] inferred that *T. sinensis* and *T. ciliata* were divergent at about 15.0 to 46.0 Mya (95%CI), implying that potentially large genetic differences could exist between them. Molecular marker studies, including the use of sequence-related amplified polymorphisms (SRAPs) and simple sequence repeats (SSRs), indicated that *T. ciliata* had a high level of population genetic differentiation (*F_st_* = 0.7924 for SRAPs and 0.35 for SSRs) and significant effects of isolation by distance (IBD) in its natural distribution in China [12,14,15]. However, these strong patterns did not occur in the population genetic structure of *T. sinensis* although it has a wider range of geographical distribution [16,17,18]. Nevertheless, these molecular studies only partially revealed genetic and evolutionary divergences between the two species.

It is well understood that comparative genomics offers a powerful approach to studying phylogeny, identifying conserved genes and gene families [19], and mapping gene collinearity among species. It also helps to characterize transposable elements, gene duplications, gene selection (positive/purifying selection), and evolution. Such analyses were widely applied to addressing many important issues concerned with evolutionary mechanisms and divergence among species [20]. Therefore, to reveal a broader molecular basis of evolutionary divergences between *T. ciliata* and *T. sinensis*, we compared the similarities and differences between the two species in their whole genome sequences.

We recently sequenced the whole genome of *T. ciliata* and inferred that *T. ciliata* and *T. sinensis* were divergent at about 6–25 Mya [21,22]. On the basis of this study, we further investigated their evolutionary divergences using comparative genomic analyses. This included analyses of the expansion and contraction of the gene family, genome collinearity, the whole genome duplication (WGD) inferred from the evolutionary rates at the synonymous site (*K*_s_) and the four-fold degenerate synonymous site (4DTv), and the LTR insertion times. The genomic difference between the two species was tested in acquiring transposable elements (TEs). Insights into the evolutionary divergence between *T. ciliata* and *T. sinensis* were gained through this combination of analyses. Moreover, similarly to Wang et al. [22], we examined the four species analyzed by Ji et al. [21], including *Arabidopsis thaliana*, *Eucalyptus grandis*, *Salix purpurea,* and *Prunus persica*, and six other angiosperm plant species (*Citrus maxima*, *Citrus reticulata*, *Populus tremula*, *Glycine max*, *Amborella trichopoda*, and *T. sinensis*) to provide more contexts for genomic comparison. This also helped view the evolutionary divergence of *T. ciliata* from more distant species of land plant.

## 2. Materials and Methods

The sample for genome sequencing was one individual *T. ciliata* var. *ciliata* (2*n* = 2x = 56) collected from Pupiao, Baoshan city, Yunnan Province, China (25.04N, 99.06E). Genomic DNA (gDNA) was isolated from young and healthy leaves of the plant using the cetyltrimethylammonium bromide (CTAB) method [23]. A high-quality de novo assembly of the *T. ciliata* genome was determined by combining Nanopore and Hi-C sequencing analyses. The genome sequences were downloaded from the CNGB Nucleotide Sequence Archive (Accession number: CNP0001985; Table S1 for website). Wang et al. [22] detailed the methods for genome sequencing and assembly, including gene prediction and annotations, estimates of gene family, and repeats or duplications. The genomic sequences of 10 other species (*A. thaliana* [24], *A. trichopoda* [25], *C. maxima* [26], *C. reticulata* [27], *E. grandis* [28], *G. max* [29], *P. persica* [30], *P. tremula* [31], *S. purpurea* [32], and *T. sinensis* [21]) were downloaded from different websites (Table S1). These species were selected partly to compare to Ji et al. [21] and partly for the availability of whole genome sequences belonging to several angiosperm tree species. However, genome sequences of *Azadirachta indica* [NCBI SRA 053330] [33] and *Xylocarpus granatum* (GenBank accession: GCA_019650275.1) in the Meliaceae family were not included because gene annotations (gff files) were not provided for downstream genomic research.

Gene predictions were analyzed using three approaches (ab initio, homology-based, and transcriptome) [22]. The specific gene family of *T. ciliata* was analyzed with clusterProfile v3.14.0 [34] for GO and KEGG enrichment. All the gene families for the eleven species were derived using OrthoFinder V2.4 software [35] and annotated with the PANTHER V15 database [36]. GO and KEGG enrichment analyses were performed on the unique gene families of *T. ciliata*. Our previous study obtained about 37,030 gene families in the *T. ciliata* genome [22]. *A. trichopoda* was set as the outgroup, and MCMCTREE in PAML v4.9i software [37,38] was used to calculate the divergence times. Timetree (http://www.timetree.org/, accessed on 12 April 2021) was used to search for the fossil times of the eleven species.

The phylogenetic relationships among the eleven species were constructed using the maximum likelihood (ML) method and IQ-TREE v1.6.11 software [39]. Construction was based on 1276 single copy genes of the eleven species. MAFFT v7.205 [40] was used to compare the sequence of each single copy gene family, and then the PAL2NAL V14 [41] program was used to convert the compared protein sequence into codon alignment. Subsequently, gBlocks v0.91b [42] was used to remove regions with poor sequence alignment or large differences. Finally, all well-aligned gene family sequences of each species were catenated to obtain a super gene. The model detection tool ModelFinder [43] provided by IQ-TREE was used for model detection, and the best model was obtained as jtt + f + i + g4. The evolutionary tree was constructed using the maximum likelihood (ML) method, with 1000 bootstraps. The ML method was used to estimate divergence times using the correlated molecular clock and JC69 model.

Based on the above gene annotations and ML phylogeny, we further conducted the following analyses from different perspectives:

(1) Expansion and contraction of gene family: From the phylogeny with estimates of divergent times and the gene family clusters in 11 species, CAFE v4.2 [44] was used to estimate the expanded or contracted numbers of gene families for each species compared to its most recent ancestor.

(2) Natural selection detection (*K*_a_/*K*_s_ (ω) test): Single-copy gene families were obtained from *P. tremula*, *S. purpurea*, *A. thaliana*, *C. reticulata*, *C. maxima*, *T. sinensis*, and *T. ciliata*. We aligned the protein sequences of each gene family with MAFFT v7.205 (parameters: --localpair—maxiterate 1000) [40], and used PAL2NAL [41] to reverse to the codon alignment sequences. We used Codeml (f3 × 4 model of codon frequencies) based on the branch-site model in PAML v4.9 [38]. Using the “Chi2” program in PAML, we performed the likelihood ratio test (LRT) for two contrasting models, model A (the foreground branch *ω* was set with positive selection, i.e., $0<\omega_{0}<1,\;\omega_{1}=1,\;\omega_{2}>1$) vs. null model *ω* (<1). The branch of *T. ciliata* was set to be foreground and all other branches were background (each background branch *ω* was set as $0<\omega_{0}<1,\;\omega_{1}=1$). When the LRT results were significant (*p* value < 0.01), the Bayesian empirical Bayes (BEB) method [45] was used to obtain the posterior probability of the positive selection site. Positive selection at the codon site was determined under a probability of greater than 0.95 (*p* > 0.95 for *ω* > 1).

(3) Genome collinearity between species: DIAMOND v0.9.29.130 [46] was used to align gene sequences between *T. ciliata* and *T. sinensis* to determine orthologous gene pairs, with an E-value of less than 10^−5^ and a C score of greater than 0.5, where the C score was filtered by JCVI v0.9.13 [47]. Next, using gene sequences (gff3 file), we determined whether orthologous gene pairs were adjacent or not on chromosomes. This step was mainly carried out with MCScanX (parameter setting: -m 5) [48]. Finally, the genes in all collinear blocks were derived and drawn by JCVI. The same method was also used to analyze the collinearity of the gene sequences of *T. ciliata* with *T. sinensis*, *G. max,* and *A. thaliana* (E-value < 10^−5^, C score > 0.5).

(4) Whole genome duplication (WGD) analysis: Mutation parameters 4DTv at the four-fold degenerate synonymous sites [49] and the *K*_s_ values at synonymous sites were estimated using WGD v1.1.1 [50]. The occurrences of the relative times of WGD within species and between species were inferred from the distributions of the 4DTv and *K*_s_ estimates.

(5) LTR insertion times: LTR_FINDER v1.07 [51] was used to search for LTR sequences and filter out redundant LTR sequences. The flanking sequences on both sides of each LTR were extracted and compared to MAFFT (parameters: --localpair—maxiterate 1000). The distance (*k*) between two sides in terms of the Kimura model [52] in MBOSS V6.6.0 [53] was calculated, and *t* = *k*/(2 × *r*) was used to estimate the divergent time with the molecular clock (*r* = 7 × 10^−9^) [54]. The Kolmogorov–Smirnov test (non-parameter statistical test) with R was used to infer the difference between *T. ciliata* and *T. sinensis* in LTR insertion times.

## 3. Results

### 3.1. Expansion and Contraction of Gene Families

**Figure 2:**
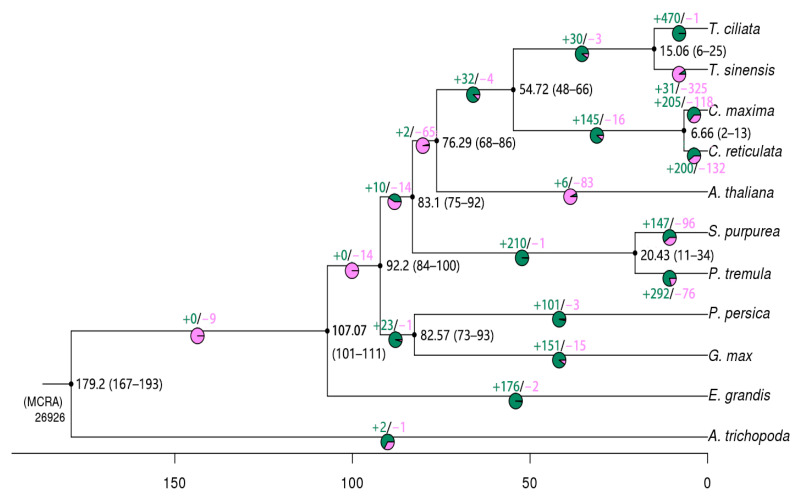
Expansion and contraction of gene families in eleven species. Pie chart sectors on each branch represent expansion of gene families in green and contraction of gene families in red. The values at each branch node are divergent times in Mya, with 95% confidence intervals in parentheses.

From the phylogeny of the eleven species (Figure S1) and the analysis of gene family clusters, the contraction and expansion of gene families were estimated for each species relative to its ancestor. Both the family-wide *p*-value and a Viterbi *p*-value of less than 5% were set to define the presence or absence of expansion or contraction of gene families. Compared with their common ancestors, *T. ciliata* had 470 expanded gene families and 1 contracted gene family (Figure 2). *T. sinensis* had 31 expanded gene families but 325 contracted gene families. PANTHER ver.14 [36] was used to annotate all gene families that were expanded or contracted in the 11 species (Table S2). All other investigated species except *A. thaliana* had more expanded than contracted gene families to different extents, compared with their corresponding common ancestors. The gene family in the *A. trichopoda* genome, the earliest divergent species in angiosperms, was slightly expanded.

GO enrichment analyses show that the expanded gene families in *T. ciliata* were involved in many biological processes (Figure S2), including metabolic and cellular processes, biological regulation, responses to stimuli, signaling, reproduction and reproductive processes, multi-organism processes, and detoxification. In terms of cellular components, the expanded genes were involved in many components, such as cells and cell parts, membranes and membrane parts, and organelles and organelle parts. The molecular functions of the expanded genes included the activities of binding, catalysis and transport, transcription regulation, determination of molecular structure, and molecular function regulation, etc. (Figure S2). The KEGG enrichment analyses show that the expanded genes were mostly associated with flavonoid biosynthesis, followed by the biosyntheses of sesquiterpenoid, triterpenoid, stilbenoid, diarylheptanoid, gingerol, and diterpenoid (Figure S2).

### 3.2. Detection of Positive Selection

Results show that twenty-five single-copy gene families were under positive selection (*p*-value of LRT test < 0.01); these are listed in Table S3. The codon sites with significant positive selection were specified in the single-copy gene family. The GO enrichment analyses indicate that these genes participated in diverse biological processes, including metabolic processes, cellular processes, responses to stimuli, organismal cellular component formation or biogenesis, and reproductive processes. In cellular components, these genes were involved in the formation of cells or cell parts, membranes or membrane parts, organelles, protein-containing complexes, membrane-enclosed lumens, cell junctions, and supramolecular complexes. In terms of molecular function, these genes were mainly involved in binding and catalytic functions (Figure S3). The KEGG analyses show that the genes under positive selection mostly participated in RNA transport, followed by the biosynthesis of glycosphingolipid, various types of N-glycan, ubiquinone and other terpenoid-quinone, and the degradation of glycosaminoglycan and other glycans (Figure S3).

### 3.3. Genome Collinearity Analysis

**Figure 3:**
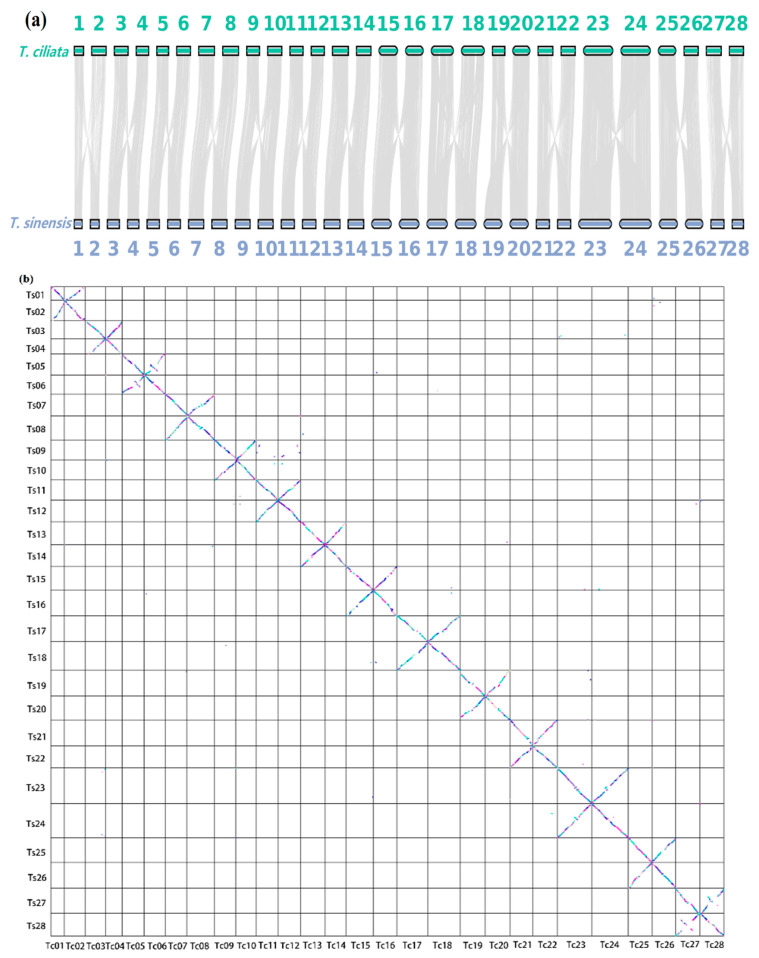
Collinearity map and dot plot of twenty-eight chromosomes shared by *T. ciliata* and *T. sinensis*: (**a**) Collinear map for each chromosome pair shared by the two species; (**b**) Dot plot of twenty-eight chromosomes shared by the two species. Symbols Tc01, Tc02, …, and Tc28 represent chromosomes 1,2, …, and 28 of *T. ciliata* genome, respectively. Symbols Ts01, Ts02, …, and Ts28 represent chromosomes 1, 2, …, and 28 of *T. sinensis* genome, respectively.

Figure 3a shows the collinearity relationship between twenty-eight shared chromosomes of the *T. ciliata* and *T. sinensis* genomes, indicating a close genetic relationship. Each chromosome pair in the two species has a strong collinear relationship. Figure 3b shows a dot matrix plot between *T. ciliata* and *T. sinensis*, exhibiting long diagonal lines across twenty-eight chromosomes but short broken lines perpendicular to the diagonals between neighbor chromosomes. However, *T. ciliata* had very weak collinearity relationships with *A. thaliana* and *G. max* (Figure S4), and most orthologous genes could have been lost or substantially divergent during the process of genome replications in each species.

### 3.4. Genome Duplications

**Figure 4:**
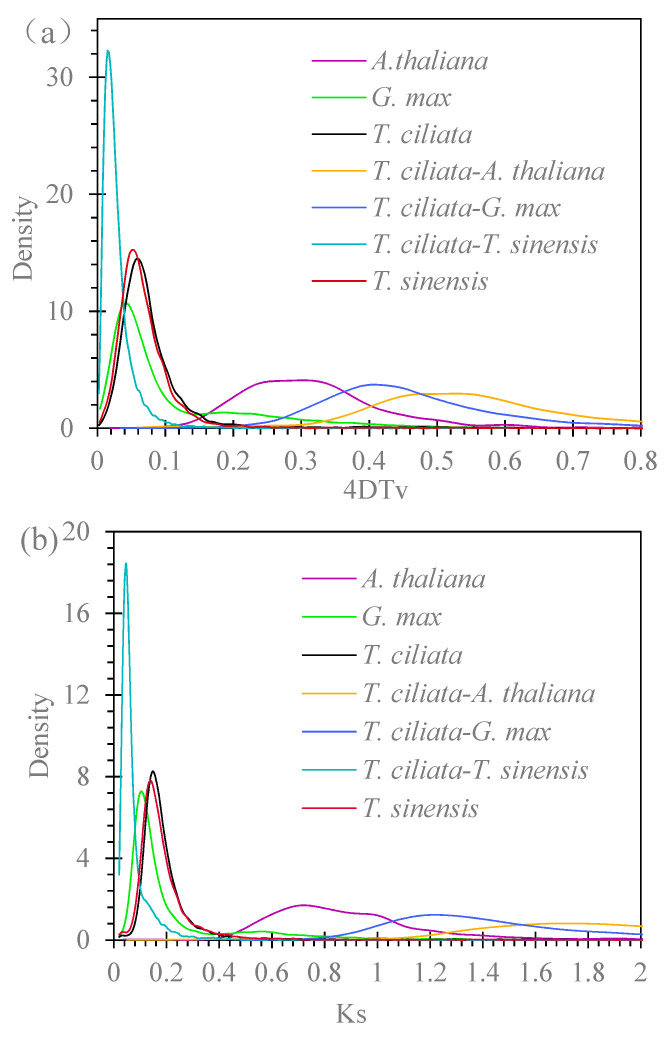
Evolutionary divergence in terms of whole genome duplication (WGD): (**a**) Distribution of 4DTv (four-fold degenerate transversion rate) within and between species genomes; (**b**) Distribution of *K*_s_ (synonymous substitution rate) within and between species genomes.

The WGD event was identified by analyzing the distributions of 4DTv and KS estimates. Figure 4a shows the density distribution of the transversion rate, 4DTv, within *T. ciliata*, *T. sinensis*, *A. thaliana,* and *G. max*, or within the pairs of *T. ciliata* with one of the other three species. Different times of WGD events occurred within and between the species. Comparison of paralogous genes in duplicated collinear blocks revealed one peak of genetic distance at 0.0585 in the *T. ciliata* genome (11450 estimates) and a peak at 0.0530 in the *T. sinensis* genome (9524 estimates). One WGD event occurred in their genomes. Comparison of orthologous genes between *T. ciliata* and *T. sinensis* (15517 estimates) reveals one peak of genetic distance at 0.0151, indicating that the WGD event occurred before speciation. The difference in 4DTv between the two peaks, about 0.0406, is greater than 0.0151, indicating a long time interval from the WGD within genomes to speciation. Comparison of the orthologous genes of *T. ciliata* with *A. thaliana* or *G. max* show that they diverged a very long time ago.

Figure 4b shows the density distribution of the synonymous substitution rate (*K*_s_) between paralogous genes within four species, or between orthologous genes within the pairs of *T. ciliata* with one of the other three species. The results indicate one *K*_s_ peak at 0.1474 in the *T. ciliata* genome (11596 estimates) and one peak at 0.1365 in the *T. sinensis* genome (9703 estimates). One *K*_s_ peak also appears at 0.0463 when comparing the orthologous genes between *T. ciliata* and *T. sinensis* (16768 estimates), supporting the view that the WGD event occurred before the speciation of these two species. The difference in *K*_s_ between the peak for the WGD and the peak for speciation is about 0.0956, also indicating a long time interval between the WGD within the genomes and speciation. Comparisons of the orthologous genes of *T. ciliata* with *G. max* or *A. thaliana* show *K*_s_ peaks at 1.204 or larger, respectively, indicating that they were divergent a very long time ago.

### 3.5. Divergence in LTR Insertion Time

**Figure 5:**
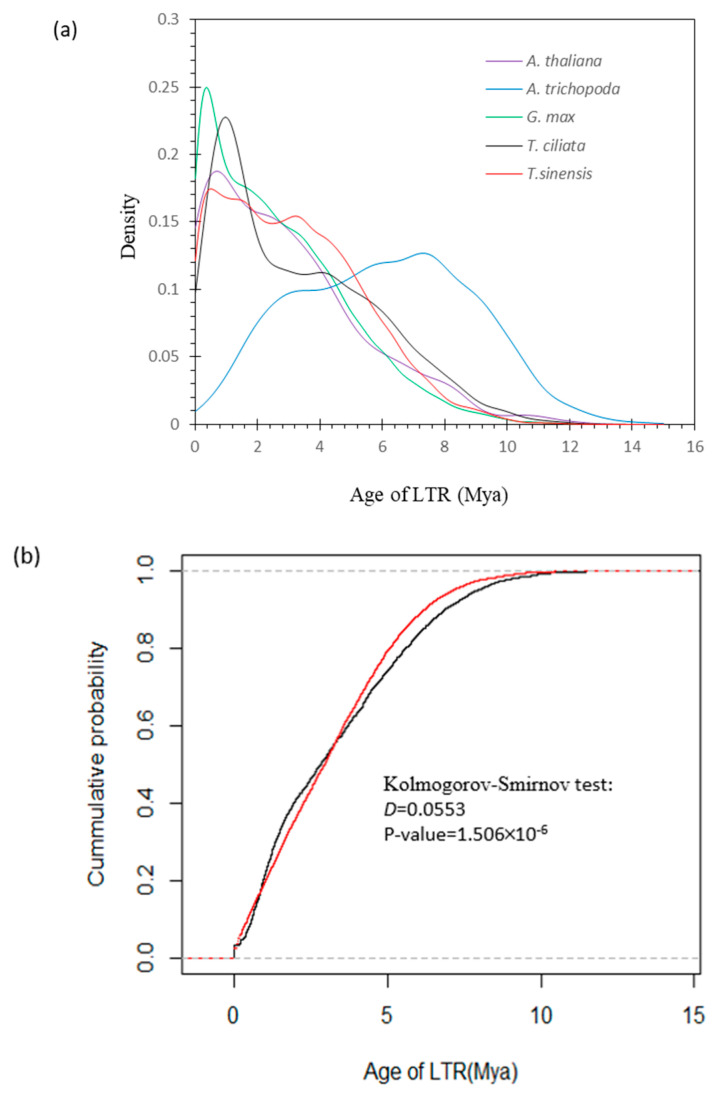
Evolutionary divergence in terms of LTR insertion times: (**a**) Distribution of the LTR insertion times in genomes of five species; (**b**) Cumulation probability of the distribution of the LTR insertion times in *T. ciliata* (black line; 4208 estimates) and *T. sinensis* (red line; 11,267 estimates) genomes.

Figure 5a shows the distributions of estimates of the LTR insertion times in five species, indicating large differences among species. Means of LTR insertion times were the longest in the genome of *A. trichopoda* (6.0478 ± 2.7766), followed by the genomes of *T. ciliata* (3.2985 ± 2.5007), *T. sinensis* (3.1516 ± 2.2097), *A. thaliana* (2.8794 ± 2.4481), and *A. max* (2.6980 ± 2.1762). LTR insertion generally occurred earlier into the *T. ciliata* genome than into the *T. sinensis* genome. A Kolmogorov–Smirnov test indicated that a significant difference existed in the distribution of LTR insertion times between *T. ciliata* and *T. sinensis* (*D* statistics = 0.0553, *p* value = 1.506 × 10^−8^, Figure 5b).

## 4. Discussion

### 4.1. Genome Comparison

In this study, we compared recently published genome sequences of *T. ciliata* with the genomes of other land plant species, with an emphasis on the genomic divergence between *T. ciliata* and *T. sinensis*. Several divergences could be concluded between the *T. ciliata* and *T. sinensis* genomes. From the assemblies of the genome sequences of these two species [21,22], *T. ciliata* had a smaller genome size than *T. sinensis* (520 vs. 596 Mb), but harbored substantially more genes than *T. sinensis* (42159 vs. 34,345 genes). Except for the annotations with KOG or a different database (Interpro), both gene annotation percentages were comparable (97.92% for *T. ciliata* vs. 97.5% for *T. sinensis*), but the *T. ciliata* genome had more genes annotated than the *T. sinensis* genome in the Nr, Swissprot, KEGG, TrEMBL, and GO databases. The main reason for such different genome sizes could result from the larger abundant repeat sequences in the *T. sinensis* genome (48.62% for *T. ciliata* vs. 64.56% for *T. sinensis*). The *T. sinensis* genome had substantially more LTR sequences (219,375,967 bp for *T. ciliata* vs. 350,754,873 bp for *T. sinensis*) and a greater proportion of LTR sequences (42.13% in the *T. ciliata* genome vs. 58.79% in the *T. sinensis* genome). The present study further indicates that the LTR sequences were more recently acquired in the *T. sinensis* genome than in the *T. ciliata* genome. This study also indicates that many gene families were lost in the *T. sinensis* genome during its speciation from their common ancestor.

Compared to the earlier analysis of *T. sinensis* genome [21], we detected only one peak in the distribution of either 4TDv or *K*_s_ estimates, each being comparable, respectively, to the first peak of the 4TDv or *K*_s_ estimates obtained by Ji et al. [21]. This consolidates the finding of one recent event of whole genome duplication. However, Ji et al.’s [21] second peak (*K*_s_ = 1.48) in Figure 5 was very small, and not detected in our analysis of the *T. sinensis* genome. Reasons for this are unknown.

### 4.2. Evolutionary Divergence and Its Implications

From TimeTree (http://www.timetree.org/, accessed on 12 April 2021), the divergent times between *T. ciliata* and *T. sinensis* covered a wide range of estimates in the literature, 7.4~48.4 Mya [55,56,57]. For instance, Muellner et al. [13] constructed a phylogeny using a few cpDNA segments and showed that the divergent time between the two species was 15.0 to 46.0 Mya. Based on 1276 single-copy gene sequences, the divergent times between *T. ciliata* and *T. sinensis* were at 15.06 (6–25 Mya) [22]. More precision estimates of divergent times between *T. ciliata* and *T. sinensis* need fossil record data of the species that are phylogenetically close to them, which could further confine the confidence intervals of divergent time estimates.

This study detected a large number of expanded gene families in the *T. ciliata* genome relative to the *T. sinensis* genome, which was likely formed in the long time process since speciation. Although these gene families were annotated (Table S3), their functions on phenotypes remain to be explored in the future. They could also be associated with the differences between the two species in phenotype or gene expression. Some of the expanded gene families and a few single-copy genes under positive selection were identified as being associated with reproduction and the reproductive process (Figures S2 and S3), implying that these genes could participate in species divergence in the reproductive system.

Generally, the genomic differences are likely associated with the differences in multiple traits, including the reproductive system and ecological adaptation. *T. ciliata* and *T. sinensis* adopt different strategies to avoid selfing and inbreeding. *T. ciliata* exhibits a predominantly outcrossing system, with selfing and inbreeding [12]. The functionally male and female flowers are disproportionally mixed and distributed in different positions on the same inflorescence, and most individuals are sexually monoecious rather than dioecious [58,59]. *T. sinensis* exhibits an outcrossing system by forming sexual diecious plants and potentially has self-incompatible genes. The more abundant LTRs and larger genome size in *T. sinensis* are likely related to the outcrossing system. This is consistent with the hypothesis that a selfing species reduces genome size and loses transposable elements [60], i.e., the characteristics of genomic selfing syndromes [61].

Difference in LTR abundance and insertion time could be related to the divergence in ecological adaptation. Although the historical cultivation could influence the present distribution of *T. sinensis* in China, a wider range distribution implies that *T. sinensis* is adaptive to more diverse habitats. Large abundant LTRs could be related to this broader adaptation of *T. sinensis*. Moreover, the genes that were present in the *T. ciliata* genome but lost in the *T. sinensis* genome are likely related to the adaptation of *T. ciliata*.

Evidence in the literature supports the significance of TEs in genome function and evolution [62,63]. The effects of LTRs on genome size variation were reported in wild barley *Hordeum spontaneum* where the copy number of BARE-1 LTR was significantly different between populations (8300~22100) and resulted in genome size variation [64]. The correlation among the BARE-1 LTR copy number, genome size, and local environmental conditions implied a linkage between the amplification of a specific TE and adaptive evolution. Wos et al. [65] also indicated that TEs including LTRs were adaptable to the environment in *Arabidopsis arenosa*. Thus, abundant LTRs in the *T. sinensis* genome could be related to its larger range size of natural distribution in China. This awaits verification through appropriate data collection.

## 5. Conclusions

In this study, we examined the evolutionary divergence between *T. ciliata* and *T. sinensis* using their whole genome sequences. These two species are closely related within the *Toona* genus of the Meliaceae family. Their divergences are summarized as follows: (1) The *T. ciliata* genome had more expanded gene families than the *T. sinensis* genome following their speciation. Many gene families were lost in the *T. sinensis* genome. (2) Compared with *T. sinensis* and other land plant species investigated, *T. ciliata* had twenty-five gene families in its genome undergoing positive selection, and these genes were involved in diverse biological processes, cellular components, and molecular functions. (3) The *T. ciliata* genome was highly collinear with the *T. sinensis* genome, but had low collinearity with the genomes of distant species. (4) The whole genome duplication occurred in *T. ciliata* and *T. sinensis* before their speciation. A longer time interval existed between the main genome duplication and speciation times. (5) LTR insertion was earlier into the *T. ciliata* genome than into the *T. sinensis* genome. These genomic differences are likely associated with differences in their reproductive systems and ecological adaptation.

## Data Availability

Links to publicly archived datasets for all genome sequences of 11 species in this study are provided in Supplementary Materials.

## Supplementary Materials

The following supporting information can be downloaded at: https://www.mdpi.com/article/10.3390/genes13101799/s1, Figure S1: A phylogeny of 11 species based on 1276 single-copy genes, Figure S2: GO and KEGG analyses for expanded gene families in *T. ciliate*, Figure S3: GO enrichment and KEGG pathway analyses of the genes under positive selection, Figure S4: Chromosome collinearity analysis, Table S1: Websites and access numbers to download genome sequences of 11 plant species used in this study, Table S2: A list of expanded gene families in *T. ciliata* and its comparison with other 10 land plant species, Table S3: Summary of the genes and gene families of *T. ciliata* under positive selection detected using branch-site model.
