# Supplementary material for: Evolutionary Divergence between Toona ciliata and Toona sinensis Assayed with Their Whole Genome Sequences
Source: Genes (Basel). 2022 Oct 5;13(10):1799. doi: 10.3390/genes13101799 (PMC9602400; doi:10.3390/genes13101799)
Supplement: Supplementary file 1 [file genes-13-01799-s001.zip › genes-1863159-supplementary.pdf]

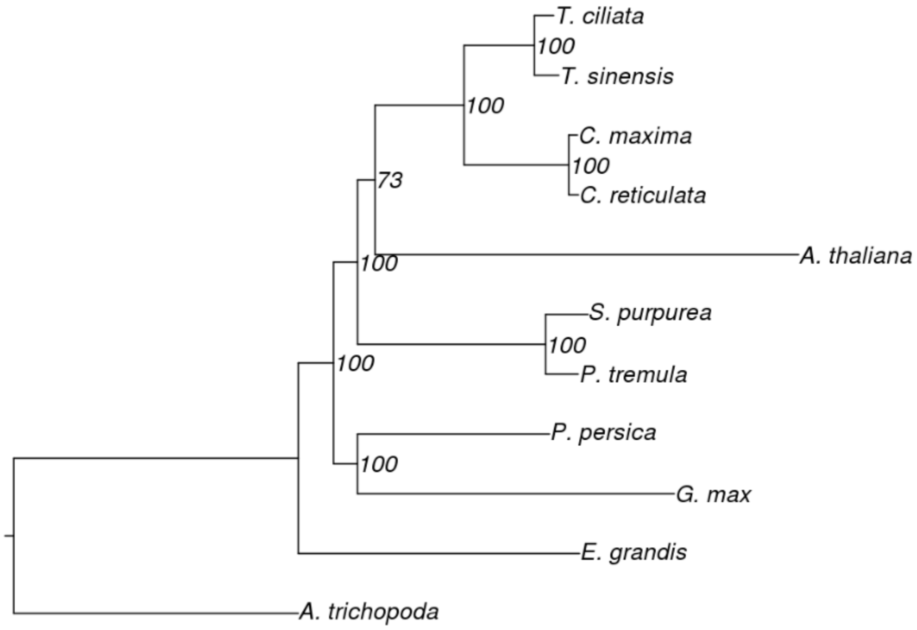

Figure S1. A phylogeny of 11 species based on 1276 single copy genes. It was constructed using IQ-TREE v1.6.11 software [1] MAFFT v7.205 [2] was used to compare the sequence of each single copy gene family, and then the PAL2NAL V14 [3] program was used to convert the compared protein sequence into codon alignment. Subsequently, gblocks v0.91b [4] was used to remove regions with poor sequence alignment or large differences. Finally, all well aligned gene family sequences of each species were catenated to obtain a super gene. Then, the model detection tool ModelFinder [5] provided by IQ-TREE was used for model detection, and the best model was obtained as jtt+f+i+g4. Then, the evolutionary tree was constructed by using the maximum likelihood (ML) method, with 1000 bootstraps. .

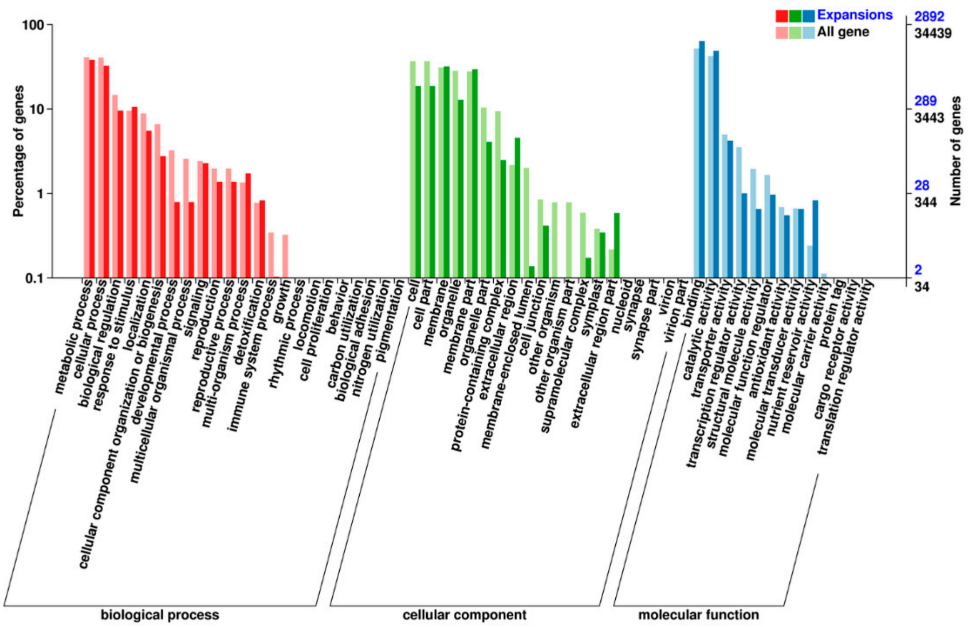

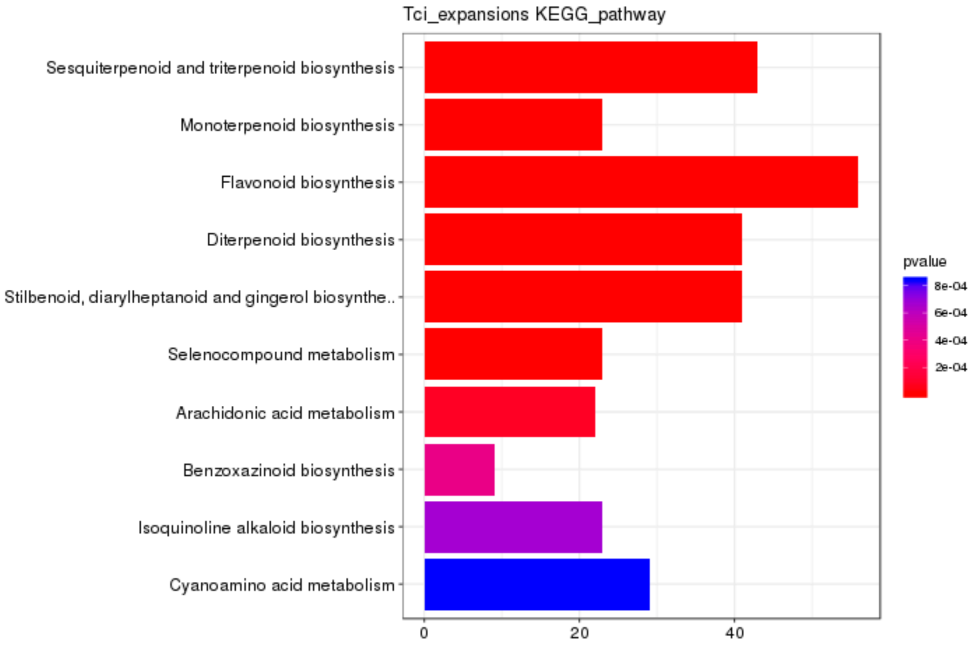

Figure S2. Go and KEGG analyses for expanded gene families in *T. ciliata***.** The upper figure is GO enrichment analysis, showing the percentages of expanded gene families in three ontologies (biological process, cellular component and molecular function). The bottom figure is KEGG pathway analysis. The ordinate is the information of pathway and the abscissa in the bar chart is the number of genes. Ten pathways with the most significant enrichment (i.e., the lowest p-values) were selected for illustrations.

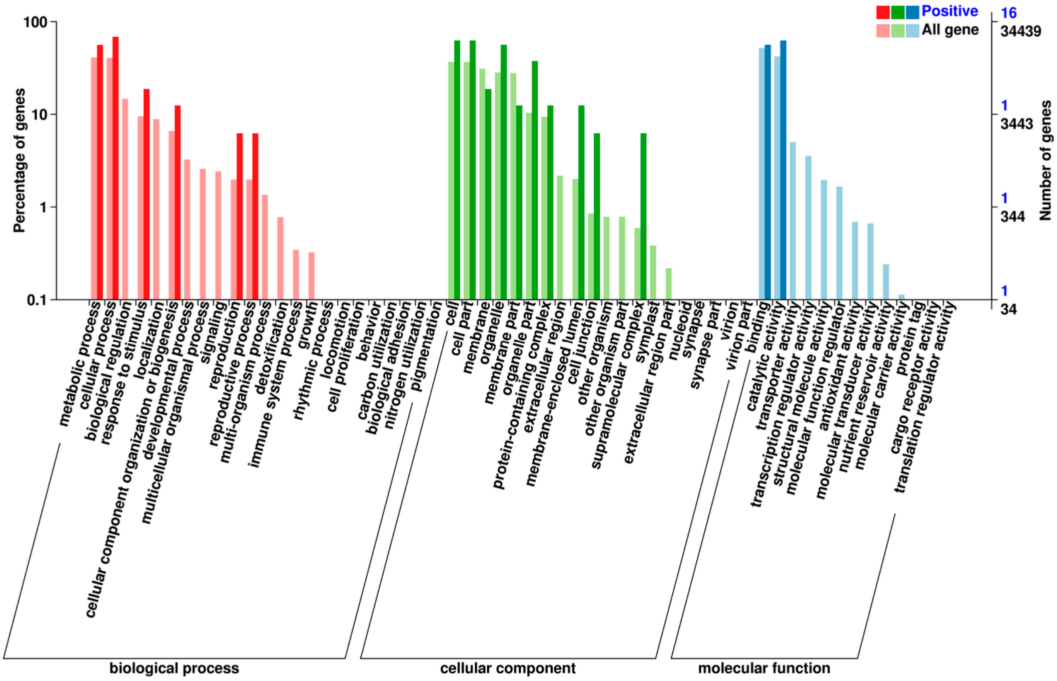

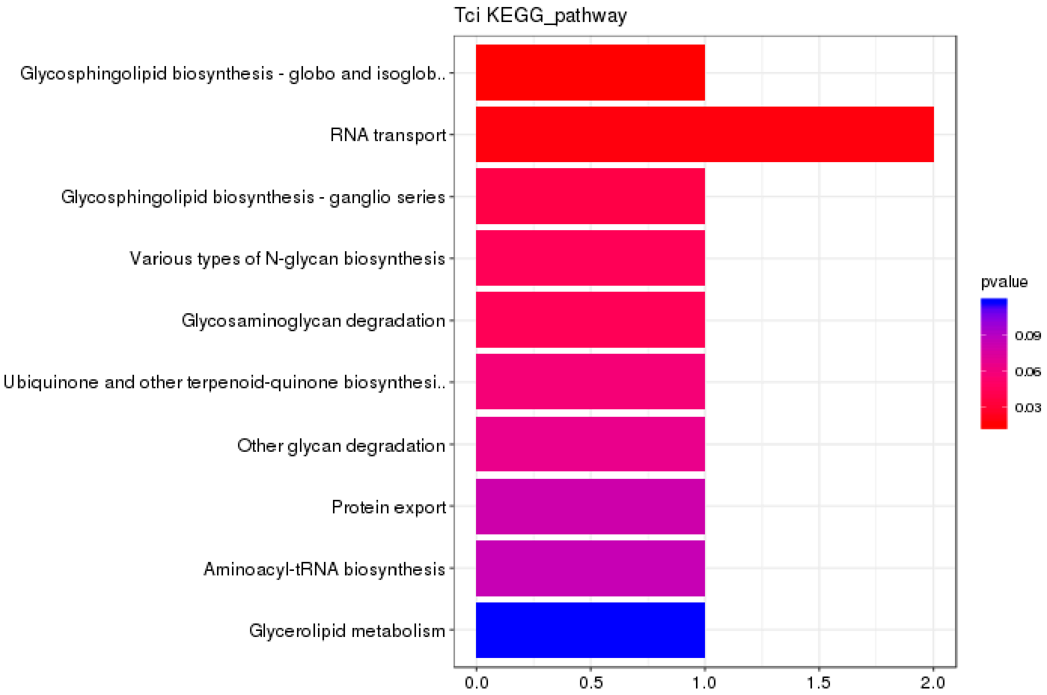

Figure S3. GO enrichment and KEGG pathway analyses of the genes under positive selection. Upper: In the biological processes, the genes with positive selection were mainly involved in metabolic process, cellular process, response to stimulus, cellular component organismal or biogenesis, reproduction and reproductive process. In terms of cellular component, these genes were involved in the components of cell and cell part, membrane and membrane part, organelle, protein-containing complex, membrane-enclosed lumen, cell junction, and supramolecular complex. In terms of molecular function, these genes were mainly involved in binding and catalytic function. Bottom: The ordinate is the information of KEGG pathway and the abscissa in the bar chart is the number of genes. Ten pathways with the most significant enrichment (i.e., the lowest p-values) were selected for illustrations.

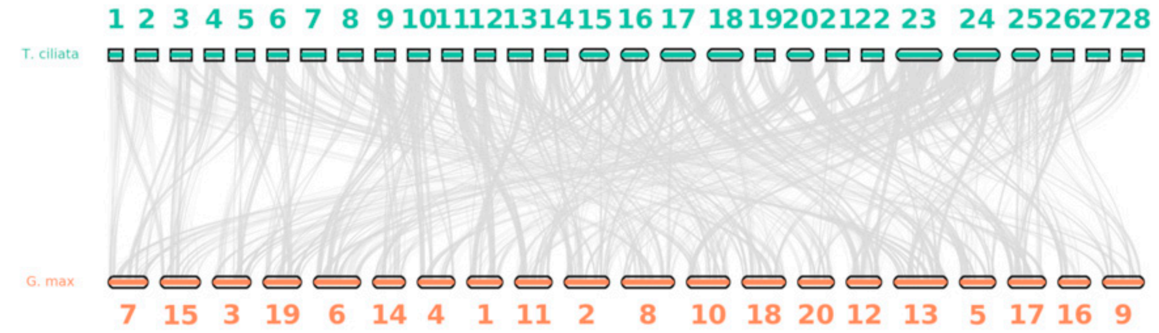

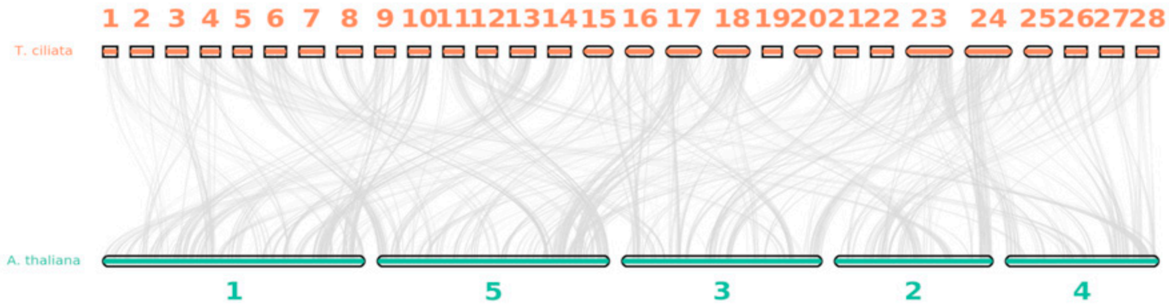

Figure S4. Chromosome collinearity analysis. The upper figure is the collinear relationship of *T. ciliata* against *G. max*, and the bottom figure is the collinear relationship of *T. ciliata* against *A. thaliana*.

Table S1. Websites and access numbers for downloading genome sequences of 11 plant species used in this study.

Species	Website	Accession number	Reference
Citrus maxima	http://citrus.hzau.edu.cn/orange/download/HWB.chromosome .fa.tar.gz	NCBI: GCA_002006925.1	[6]
Populus tremula	http://128.192.158.63/index.php/databases/spta-717-genome	ENA: PRJEB23585	[7]
Salix purpurea	https://phytozome-next.jgi.doe.gov/info/Spurpurea_v5_1	JGI	[8]
Eucalyptus grandis	https://phytozome.jgi.doe.gov/pz/portal.html#!info?alias=Org_ Egrandis	NCBI: GCA_016545825.1	[9]

Amborella trichopoda	https://phytozome.jgi.doe.gov/pz/portal.html	NCBI: GCA_000471905.1	[10]
Prunnus persica	http://genome.jgi.doe.gov/pages/dynamicOrganismDownload.jsf?organism=Ppersica	NCBI: GCA_000346465.2	[11]
Citrus reticulata	http://citrus.hzau.edu.cn/orange/download/index.php	NCBI: GCA_003258625.1	[12]
Arabidopsis thaliana	ftp://ftp.ensemblgenomes.org/pub/plants/release-45/fasta/arabidopsis_thaliana	NCBI: GCA_000001735.2	[13]
Glycine max	https://phytozome.jgi.doe.gov/pz/portal.html#!info?alias=Org_Gmax	NCBI: GCA_000004515.5	[14]
Toona sinensis	https://db.cngb.org/search/project/CNP0000958/	CNGB: CNP0000958	[15]
Toona ciliata	https://db.cngb.org/search/project/CNP0001985/	CNGB: CNP0001985	[16]

Table S2. A list of expanded gene families in *T. ciliata* and its comparison with other 10 lant plant species.

Orthogroup	Ath	Atr	Cma	Cre	Egr	Gma	Pep	Ptr	Spu	Tci	Tsi	Total	AnnoGeneNum(PANTHER_ID)	PANTHER_Description
OG0000002[Tci+]	21	0	22	38	0	1	5	1	4	36	0	128	128[PTHR23155:SF1044]	OS12G0491200 PROTEIN
OG0000004[Tci+]	8	6	6	7	7	17	7	30	11	14	7	120	120[PTHR24068:SF346]	BNAA01G32320D PROTEIN
OG0000007[Tci+]	2	0	21	25	1	0	8	24	9	20	0	110	110[PTHR31170:SF13]	BNAC04G53230D PROTEIN
OG0000009[Tci+]	0	0	8	5	40	2	22	2	11	17	0	107	107[PTHR27005:SF367]	SUBFAMILY NOT NAMED
OG0000011[Tci+]	11	0	7	6	5	15	7	15	12	10	4	92	92[PTHR11426:SF198]	SUBFAMILY NOT NAMED
OG0000013[Tci+]	0	0	15	16	31	4	7	3	4	10	0	90	90[PTHR31225:SF111]	TERPENE SYNTHASE 2-RELATED
OG0000022[Tci+]	0	0	1	2	6	1	6	22	35	7	0	80	80[PTHR32444:SF130]	GLYCOPROTEIN S13, PUTATIVE-RELATED
OG0000023[Tci+]	0	0	18	24	0	0	11	6	4	15	0	78	78[PTHR48063:SF13]	RECEPTOR-LIKE PROTEIN 12 ISOFORM X1
OG0000027[Tci+]	0	0	9	10	0	0	0	23	11	23	0	76	76[PTHR31170:SF9]	PROTEIN, PUTATIVE (DUF247)-RELATED

Table S3. Summary of the genes and gene families of *T. ciliata* under positive selection detected using branch-site model.

Gene	Branch p-value	Sites	Gene family
Tci0G012920	0.000001812	516,S,0.991** 517,F,0.994**	OG0010586
Tci02G003970	0.009595759	203,M,0.999** 204,E,0.999**	OG0010741
Tci26G007640	0.004636312	845,Q,0.987* 846,E,0.996**	OG0010779
Tci25G015770	0.000001611	189,D,0.998** 191,L,0.976* 299,E,1.000** 301,E,0.995**	OG0011971
Tci18G007370	0	270,E,0.990* 274,L,0.998** 277,R,0.983* 279,R,0.995** 283,L,1.000** 284,S,0.998** 288,E,0.997** 290,R,0.997** 291,R,0.993**	OG0012039
Tci26G008500	0.007148477	81,A,0.989*	OG0012120
Tci24G009130	0	791,S,0.995** 793,S,0.998** 794,L,0.994** 797,N,0.974* 804,R,0.995** 805,E,0.973* 807,A,0.999** 811,Q,0.999** 812,K,0.982* 813,E,0.999**	OG0012180
Tci24G012060	0	273,C,0.990** 276,A,0.988* 277,D,0.996**	OG0012181
Tci05G006200	0	221,A,0.991** 222,Y,0.989* 223,I,0.999** 224,T,0.992** 225,K,0.980* 226,N,1.000** 233,Q,0.997** 236,T,0.980* 245,Q,0.979*	OG0012339
Tci16G006340	0	2,L,0.957* 8,Q,1.000** 9,M,0.989* 15,V,0.985* 17,L,0.973* 18,R,0.996** 148,R,0.997**	OG0012457
Tci22G012170	0.008371652	837,K,0.992**	OG0012509
Tci03G000720	0	384,T,0.963* 395,D,0.996** 396,Q,0.999** 398,L,0.978* 400,A,0.997** 401,S,0.999** 402,S,0.997** 403,M,0.991** 408,W,0.999**	OG0012568
Tci08G010320	0	1568,Q,0.991** 1655,L,0.960* 1659,L,0.962* 1661,G,0.958* 1662,Y,0.988*	OG0013415
Tci28G008070	0.009422131	1307,K,0.951*	OG0013543
Tci16G002340	0.000000002	4,E,0.966* 6,H,0.997**	OG0013575
Tci20G008110	0.000025704	2,E,0.997** 3,D,0.999**	OG0013722
Tci25G014390	0	94,E,0.999** 96,A,0.998** 98,E,0.997** 431,G,0.999** 433,R,0.997** 434,E,0.999** 437,E,0.999** 439,H,0.999** 440,H,1.000** 442,V,0.999** 443,T,0.972* 447,V,0.999** 449,A,0.999** 450,A,0.999** 453,A,0.997** 456,Y,0.999** 457,I,0.973* 461,T,0.999** 463,R,0.984* 467,A,0.996** 470,K,0.999** 473,M,0.967* 474,E,1.000** 475,I,1.000** 673,T,0.958* 927,S,1.000** 953,M,0.961* 955,A,0.999**	OG0013759

Tci23G023080	0	166,C,0.970* 584,C,0.998** 585,L,0.980* 586,Q,0.962* 589,H,0.999** 591,F,0.999** 592,G,0.998** 593,K,0.999** 596,R,0.992**	OG0013832
Tci20G014370	0.000403636	140,A,0.952* 179,D,0.999** 180,Y,0.999** 181,I,0.992** 182,H,0.999** 183,F,0.997** 189,A,1.000**	OG0013939
Tci12G009830	0.007667313	3,S,0.968*	OG0014682
Tci10G010830	0	86,I,0.961* 217,I,0.959* 222,D,0.970* 224,N,0.999** 225,T,0.996** 227,C,0.973* 228,W,0.998** 229,T,0.999** 230,C,0.968* 231,L,0.954* 232,F,0.986* 235,Y,0.991** 237,K,0.996** 238,A,0.985* 242,I,0.990* 248,L,0.988* 249,G,0.981* 707,M,0.990** 796,R,0.997** 797,W,0.998** 798,G,0.967*	OG0014810
Tci19G010360	0.000120128	25,N,0.990*	OG0014915
Tci22G004720	0.000000105	200,S,0.950* 452,D,1.000** 461,G,0.997** 462,E,0.999**	OG0014969
Tci11G009910	0.000000391	66,S,0.967* 101,P,0.987*	OG0015668
Tci04G011030	0.000006037	9,I,0.960* 12,R,0.989* 21,D,0.988*	OG0016558

*: a significant level of greater than 0.95; **: a significant level of greater than 0.99.

References

1. Nguyen, L.T.; Schmidt, H.A.; Von Haeseler, A.; Minh, B.Q. IQ-TREE: A fast and effective stochastic algorithm for estimating maximum-likelihood phylogenies. *Mol. Biol. Evol.* **2015**, *32*, 268–274. https://doi.org/10.1093/molbev/msu300.
2. Katoh, K.; Asimenos, G.; Toh, H. Multiple alignment of DNA sequences with MAFFT. *Methods Mol. Biol.* **2009**, *537*, 39–64. https://doi.org/10.1007/978-1-59745-251-9_3.
3. Suyama, M.; Torrents, D.; Bork, P. PAL2NAL: Robust conversion of protein sequence alignments into the corresponding codon alignments. *Nucleic Acids Res.* **2006**, *34* (Suppl 2), W609–W612. https://doi.org/10.1093/nar/gkl315.
4. Talavera, G.; Castresana, J. Improvement of phylogenies after removing divergent and ambiguously aligned blocks from protein sequence alignments. *Syst. Biol.* **2007**, *56*, 564–577. https://doi.org/10.1080/10635150701472164.
5. Kalyaanamoorthy, S.; Minh, B.Q.; Wong, T.K.; Haeseler, V.A.; Jermiin, L.S. ModelFinder: Fast model selection for accurate phylogenetic estimates. *Nat. Methods* **2017**, *14*, 587. https://doi.org/10.1038/nmeth.4285.
6. Wang, X.; Xu, Y.; Zhang, S.; Cao, L.; Huang, Y.; Cheng, J.; Wu, G.; Tian, S.; Chen, C.; Liu, Y.; et al. Genomic analyses of primitive, wild and cultivated citrus provide insights into asexual reproduction. *Nat. Genet.* **2017**, *49(5)*, 765–772. https://doi.org/10.1038/ng.3839.
7. Lin, Y.C.; Wang, J.; Delhomme, N.; Schiffthaler, B.; Sundström, G.; Zuccolo, A.; Nystedt, B.; Hvidsten, T.R.; de la Torre, A.; Cossu, R.M.; et al. Functional and evolutionary genomic inferences in *Populus* through genome and population sequencing of American and European aspen. *Proc. Natl. Acad. Sci. U.S.A.* **2018** *,115(46)*, E10970–E10978. https:/doi.org/10.1073/pnas.1801437115.
8. Zhou, R.; Macaya-Sanz, D.; Carlson, C.H.; Schmutz, J.; Jenkins, J.W.; Kudrna, D.; Sharma, A.; Sandor, L.; Shu, S.; Barry, K.; et al. A willow sex chromosome reveals convergent evolution of complex palindromic repeats. *Genome Biol.* **2020**, *21(1)*, 38. https:/doi.org/10.1186/s13059-020-1952-4.
9. Bartholomé, J.; Mandrou, E.; Mabiala, A.; Jenkins,J.; Nabihoudine, I.; Klopp,C.; Schmutz,J.; Plomion,C.; Gion,J.-M. High-resolution genetic maps of *Eucalyptus* improve *Eucalyptus grandis* genome assembly. *New Phytol.* **2015**, *206(4)*, 1283–1296. https:/doi.org/10.1111/nph.13150
10. Amborella Genome Project. The *Amborella* genome and the evolution of flowering plants. *Science* **2013**, *342(6165)*, 1241089. doi:10.1126/science.1241089.

11. International Peach Genome Initiative; Verde, I.; Abbott, A.G.; Scalabrin, S.; Jung, S.; Shu, S.; Marroni, F.; Zhebentyayeva, T.; Dettori, M.T.; Grimwood, J.; et al. The high-quality draft genome of peach (*Prunus persica*) identifies unique patterns of genetic diversity, domestication and genome evolution. *Nat. Genet.* **2013**, *45(5)*, 487–494.https:/doi.org/10.1038/ng.2586.
12. Wang, L.; He, F.; Huang, Y.; He, J.; Yang, S.; Zeng, J.; Deng, C.; Jiang, X.; Fang, Y.; Wen, S.; et al. Genome of wild mandarin and domestication history of mandarin. *Mol. Plant* **2018**, *11(8),* 1024–1037. https://doi.org/10.1016/j.molp.2018.06.001.
13. Sloan, D.B.; Wu, Z.; Sharbrough, J. Correction of persistent errors in Arabidopsis reference mitochondrial genomes. *Plant Cell* **2018**, *30(3)*, 525–527. doi:10.1105/tpc.18.00024.
14. Schmutz, J.; Cannon, S.B.; Schlueter, J.; Ma, J.; Mitros, T.; Nelson, W.; Hyten, D.L.; Song, Q.; Thelen, J.J.; Cheng, J.; et al. Genome sequence of the palaeopolyploid soybean. *Nature* **2010**, *463(7278)*, 178–183. https:/doi.org/10.1038/nature08670.
15. Ji, Y.-T.; Xiu, Z.-H.; Chen, C.-H.; Wang, Y.; Yang, J.-X.; Sui, J.-J.; Jiang, S.-J.; Wang, P.; Yue, S.-Y.; Zhang, Q.-Q.; et al. Long read sequencing of *Toona sinensis* (A. Juss) Roem: A chromosome-level reference genome for the family Meliaceae. *Mol. Ecol. Resour.* **2021**, *21*, 1243–1255. https://doi.org/10.1111/1755-0998.13318.
16. Wang, X.; Xiao, Y.; He, Z.H.; Li, L.L.; Song, H.Y.; Zhang, J.J.; Cheng, X.; Chen, X.Y.; Li, P.; Hu, X.S. A chromosome-level genome assembly of *Toona ciliata* (Meliaceae). *Genome Biol. Evol* **2022**, *14*, evac121. https://doi.org/10.1093/gbe/evac121.
